# The purple line as a measure of labour progress: a longitudinal study

**DOI:** 10.1186/1471-2393-10-54

**Published:** 2010-09-16

**Authors:** Ashley Shepherd, Helen Cheyne, Susan Kennedy, Colette McIntosh, Maggie Styles, Catherine Niven

**Affiliations:** 1 Department of Nursing and Midwifery, University of Stirling, Stirling, FK9 4LA, UK; 2 Nursing, Midwifery and Allied Health Professions Research Unit, University of Stirling, Stirling, UK; 3 Forth Valley NHS Trust, Stirling Royal Infirmary, Stirling, FK8 2AU, UK

## Abstract

**Background:**

Vaginal examination (VE) and assessment of the cervix is currently considered to be the gold standard for assessment of labour progress. It is however inherently imprecise with studies indicating an overall accuracy for determining the diameter of the cervix at between 48-56%. Furthermore, VEs can be unpleasant, intrusive and embarrassing for women, and are associated with the risk of introducing infection. In light of increasing concern world wide about the use of routine interventions in labour it may be time to consider alternative, less intrusive means of assessing progress in labour. The presence of a purple line during labour, seen to rise from the anal margin and extend between the buttocks as labour progresses has been reported. The study described in this paper aimed to assess in what percentage of women in labour a purple line was present, clear and measurable and to determine if any relationship existed between the length of the purple line and cervical dilatation and/or station of the fetal head.

**Methods:**

This longitudinal study observed 144 women either in spontaneous labour (n = 112) or for induction of labour (n = 32) from admission through to final VE. Women were examined in the lateral position and midwives recorded the presence or absence of the line throughout labour immediately before each VE. Where present, the length of the line was measured using a disposable tape measure. Within subjects correlation, chi-squared test for independence, and independent samples t-test were used to analyse the data.

**Results:**

The purple line was seen at some point in labour for 109 women (76%). There was a medium positive correlation between length of the purple line and cervical dilatation (r = +0.36, n = 66, P = 0.0001) and station of the fetal head (r = +0.42, n = 56, P < 0.0001).

**Conclusions:**

The purple line does exist and there is a medium positive correlation between its length and both cervical dilatation and station of the fetal head. Where the line is present, it may provide a useful guide for clinicians of labour progress along side other measures. Further research is required to assess whether measurement of the line is acceptable to women in labour and also clinicians.

## Background

There are a number of ways of measuring progress in labour including assessment of contractions, descent and position of the fetal head by abdominal palpation and assessment of cervical dilatation by vaginal examination (VE). VE is currently considered to be the gold standard for assessment of labour progress [1], however there are a number of problems associated with this examination. Studies to assess the accuracy of the digital examination of the cervix are limited but those that do exist suggest that the assessment is imprecise. Some studies [2,3] have used hard cervical models in which the cervix is fixed in position to measure the accuracy of midwives and obstetricians in measuring cervical dilatation. They reported an overall accuracy for determining the exact cervical diameter of between 48.6% and 56.3% which improved to between 89.5% and 91.7% when an error of ± 1 cm was allowed. Both studies noted that the accuracy of the examination decreased as cervical dilatation increased. A recent study which utilised more realistic soft cervical models found that only 19% of cervical dilatation measurements were accurate [4].

Guidelines from the Royal College of Midwives [5] suggest that VEs should be carried out by the same midwife throughout labour to reduce inter-observer variability and inaccuracy. A recent study [1], noted that in a group of 508 women, two clinicians differed in dilatation measurements by 2 cm or more in 11% of occasions. An inconsistent finding between examiners has been noted to cause distress in women and has resulted in them losing confidence in their health care providers [6]. Interestingly, one study [2] noted that where an error in measurement was recorded, the direction of the error was inconsistent i.e. individuals did not always over or underestimate the measurement. This is of concern as the decisions made regarding progress and interventions in labour often rely on measurement of cervical dilatation [7]. Such interventions include artificial rupture of membranes, use of oxytocin and even caesarean section. This 'cascade of intervention', where one intervention in labour leads to another has been described in the literature [8-10]. In order to ensure women are not subjected to unnecessary intervention, it is essential that accurate assessment of progress in labour takes place.

In addition to inconsistency, VEs can be unpleasant, intrusive and embarrassing for women [6,11,12]. Women with a history of fetal loss or previous gynaecological surgery have reported feeling distressed during such an examination [13]. It has also been suggested that there is an association between post traumatic stress disorder and history of sexual violence, which can then impact upon a woman undergoing VE [14]. These women describe feelings of powerlessness and physical pain. This is supported by a recent study which reported that women who experienced greatest stress during VEs were women with a history of sexual violence and post traumatic stress disorder [15].

There is a reported link between the numbers of VEs a woman has and the risk of pueperal sepsis [16-19]. Currently, in the USA, 5.5% of vaginal deliveries and 7.4% of caesarean deliveries are complicated by postpartum infections [20]; the greater the number of VEs associated with an increased risk of genital tract infection.

Due to the problems associated with VEs noted above, guidelines have recommended their restricted use. For example, the National Institute for Health and Clinical Excellence (NICE) Intrapartum guidelines recommend that VEs should not be routinely performed and that women should be 'offered' a VE every four hours in the first stage of labour [21]. Several initiatives, including the 'All Wales Pathway' and 'Keeping Childbirth Natural and Dynamic' (KCND Scotland) have been developed in an attempt to limit interventions in normal labour and encourage a more holistic and less prescriptive approach to labour care [22,23]. These pathways include guidance on VEs; both seeking to limit the number and frequency. A recent trial examining diagnosis of labour found that although the mean number of VEs was three, this ranged from 0 to 11 VEs (the average length of labour was eight hours) [24]. The WHO [25] recommends that the number of VEs should be limited to those which are strictly necessary and ideally this should be the one examination to establish active labour.

It is debatable whether VEs are indeed the most appropriate method for assessing progress in labour. However, they have become so routine in labour care that they are no longer seen as an intervention [26]. Walsh [27] suggests that "routine repeated VEs in normal labour should be abandoned until research establishes their appropriate place". Over the last 10 years there has been an increased interest in reducing intervention in normal labour, with recommendations from NICE for further research into the number, frequency and risk associated with VEs [21]. In light of increasing concern world wide about the use of routine intervention in labour it is time to consider alternative, less intrusive means of assessing progress in labour.

Prior to the 1970s, midwives placed more emphasis on alternative methods of assessing labour progress [28]. These included monitoring the patterns of uterine contractions and measuring descent and flexion of the fetal head by abdominal palpation. However, increasing medicalisation of labour throughout the 1980s has meant that generations of midwives have prioritised the VE as the primary method of assessing labour progress. Over reliance on the VE may have influenced the ability of midwives to develop and retain confidence in other methods of assessment [28].

In the attempt to minimise intervention in labour, there has been increased interest in assessing progress through non-invasive means. Some studies [29,30] have described behaviours and vocalisations indicative of the second stage of labour. One study [30] concluded that the sounds made by women in labour can provide information about the nature of pain that the woman is experiencing and thus the stage of labour reached. It would seem reasonable to suggest that midwives need to be alert to the changing behaviours and vocalisations of women in labour, as changes may be indicative of progress. However, assessing labour in this way requires the midwife to have known the women she is caring for before labour begins, and this is not as yet an option for midwifery care in many areas. It is clear that further research in this area is needed if assessment of these behaviours is to prove useful in the evaluation of labour progress.

Whilst these observational measurements may be non-invasive, Hobbs [31] suggests that what women want is a non-invasive yet objective measurement to know how far they have progressed in labour and that midwives also want to be able to give an accurate account of how labour is progressing. With the increase in interest in supporting normal labour and the drive to reduce unnecessary intervention, less invasive methods of assessing progress in labour should be investigated. However, any methods of assessing progress in labour, either as an alternative or as an adjunct to VE, must be at least as reliable.

Byrne and Edmonds [32] were the first to document the appearance of a line of red/purple discolouration seen to arise from the anal margin and extend between the buttocks reaching the nape of the buttocks (just below the sacrococcygeal joint where the coccyx begins to curve inwards) at the onset of the second stage of labour. They conducted a small study with 48 women in spontaneous labour and noted that the purple line was seen on 89% of occasions, was completely absent in five women (10%), and initially absent in three (6%). There was a significant correlation between the length of the purple line and cervical dilatation and the station of the fetal head. These findings suggested that assessment of the purple line has the potential to provide a reliable non-invasive measure of labour progress; however, this study was too small to draw definite conclusions.

Although the cause is not known, it has been suggested that the appearance of the purple line may be due to vasocongestion at the base of the sacrum [32]. Byrne and Edmonds suggested that this congestion possibly occurs because of intrapelvic pressure as the fetal head descends which may account for the correlation between station of the fetal head and purple line length. The rhombus of Michaelis is a kite shaped area over the lower back that includes the lower lumbar vertebrae and sacrum. It is believed that this area of bone moves backwards during advanced labour, pushing out the wings of the ilea and increasing the pelvic diameter. These normal changes to the shape of the pelvis may offer an alternative explanation to the appearance of the purple line.

A number of midwifery text books [33,34] refer to the purple line as a means of assessing the progress of labour but state that there is little evidence to support its use. In a qualitative study [35] using one focus group and a number of interviews with one experienced midwife, the 'red line' was described as an external sign that labour was progressing and its length was between 1-2 cm in the latent/prelabour stage and 4-5 cm in the early active labour stage. Hobbs [31] described her observations of the purple line in an anecdotal paper and noted that in the women she cared for it was a "reliable indicator of progress", however no evidence base was provided to justify this conclusion. Hobbs' article [31] was cited as one of the key midwifery papers to be published in the last ten years due to the debate that followed of alternative, less invasive ways, of assessing women's progress in labour [36].

The primary aim of this study was to assess in what percentage of women in labour a purple line was present, clear and measurable and to determine if any relationship existed between the length of the purple line and cervical dilatation or descent of the fetal head. Secondary outcome measures included whether the presence and length of the line differed in groups due to parity, type of labour (spontaneous or induction), baby's birth weight or length of time in labour.

## Methods

This longitudinal observational study was conducted over a 3 month period in one NHS hospital in Scotland with approximately 3,300 births per year. Primiparous and multiparous women were eligible if they were aged 16 or over with uncomplicated, singleton pregnancies and were admitted at term either in spontaneous labour or for induction of labour.

A power calculation (based on the findings of Byrne and Edmonds [32]) was conducted for the primary outcome of presence or absence of the purple line at any point during labour. Using a one-sided binomial test, for the purple line to be present in 90% of observations (at an 80% confidence and an alpha level of 0.05) a minimum sample of 84 women was required. In order to ensure that correlational analysis was adequately powered, we aimed to collect data on a minimum of 100 women and to collect data throughout labour.

All potentially eligible women were given information about the study at their 34-36 week antenatal appointment. On admission to hospital, study eligibility was checked by the admitting midwife. If eligible, and the midwife judged that the women was not too distressed, women were given a further explanation of the study and asked to give written consent. Immediately before routine admission observations, women who consented were assessed whilst lying in the lateral position. The total distance from the anal margin to the nape of the buttocks was measured using a disposable tape measure. The presence or absence of a purple line was noted and, if present, the length of the line was measured (in centimetres) and then recorded as a percentage of the total distance. For women admitted for induction of labour the first data collection point was prior to induction. Two midwives were present for each recording of the line length. One of these midwives then carried out a VE where cervical dilatation and station of the fetal head were recorded.

### Analysis

All data was entered onto an SPSS 15.0 data base. Analysis was conducted using a chi-squared test for Independence for presence of line and type of labour and parity. An independent samples t-test was conducted to compare the birth weight and length of labour between women who had a line present and those with no line. The relationship between length of the purple line (recorded as a percentage of the total distance from anal margin to nape of buttocks) with cervical dilatation and station of the fetal head was analysed using a within subjects correlation analysis [37]. This analysis explored whether changes in line length were paralleled by changes in either cervical dilatation or station of the fetal head. For this reason, at least two measurements for each variable were required and therefore women with only one of these variables recorded were excluded from this analysis.

### Ethics

Approval was granted by Tayside, Fife and Forth Valley NHS Ethics Committee, (Ref 08/S1402/17).

## Results

Three hundred and fifty women were invited to participate, of which, 189 consented (54%). Of those who agreed to participate, the data collection commenced but was incomplete for 21 women, 8 women withdrew from the study during labour for unknown reasons and for 16 women the data was missing. Complete data for cervical dilatation and length of line was recorded throughout labour for 144 women and it is the data from these women that is reported here. The baseline characteristics of this group are shown in table 1.

**Table 1:** Baseline Characteristics of Sample (n = 144)

Age (yrs)	Gestation at birth (weeks)	Birth Weight (grams)	Ethnicity Caucasian	Primiparous	Spontaneous Labourers	Delivery			
						SVD	Forceps	Ventouse	Emergency C.Section
Median	Median	Median	N	N	N	N	N	N	N
(SD)	(SD)	(SD)	(%)	(%)	(%)	(%)	(%)	(%)	(%)
30 (6.1)	40 (1.3)	3540 (527.4)	140 (97)	83 (58)	112 (78)	108 (75)	17 (12)	6 (4)	13 (9)

The number of VEs carried out on each woman in the group (n = 144) ranged from one to seven (Mean 2.9, SD 1.5). In total, for this group, midwives recorded both cervical dilatation and presence or absence of the purple line on 413 occasions. The line was visible on 232 (56.2%) occasions, was absent on 172 (41.6%) occasions, and was either not recorded or missing on nine occasions (2.2%).

The line was present at some point during labour for 109 women (76%). The relationship between the length of the purple line and both cervical dilatation and station of the fetal head was investigated using a within subjects correlation analysis. Analysis only included those women who had 2 or more measurements of the length of line, cervical dilatation and/or station of the fetal head. There was a medium positive correlation between length of the line and cervical dilatation r = +0.36, n = 66, P = 0.0001 and also a medium positive correlation between length of the line and station of the fetal head, r = +0.42, n = 56, P < 0.0001.

The purple line was significantly more likely to be present in women in spontaneous labour (n = 90, 80%) when compared with those women admitted for induction of labour (n = 19, 59%), X^2 ^(df = 1, n = 144) = 4.9, p = 0.03, phi = -0.20. However, no significant association between parity and presence of line was found, X^2 ^(df = 1, n = 144) = 0.43, p = 0.51, phi = -0.07.

An Independent samples t-test was conducted to compare the birth weight and time in labour of those women who had a line present at some point in their labour and those women who had no line at any time. There was no significant difference in birth weight for those women with a line (mean 3581 grams, SD 527) and those without a line (mean 3437 grams, SD 520; t(142) = 1.4, p = 0.16). There was also no significant difference in the length of time in labour for those women who had a visible line (8.5 hours, SD 4.5) and those without a line (7.1 hours, SD 4.5; t(142) = 1.6, p = 0.11).

For women admitted in spontaneous labour (n = 112), 303 VEs and measurements of the line were noted throughout labour. The number of VEs carried out and the percentage of examinations where the line was present (at different cervical dilatations) is shown in table 2. For example, midwives carried out 22 VEs and line examinations where the cervical dilatation was 1-2 cm and the line was present during 6 (27.3%) of these examinations. This table illustrates an increase in the percentage of examinations where a purple line was present as cervical dilatation increased. Also the mean length of the line increased from 5.3 cm (SD = 3.1) when the cervix was 1-2 cm dilated to 9.6 cm (SD = 2.1) when the cervix was 9-10 cm dilated. For women admitted for induction of labour (n = 32), 105 VEs and measurements of the line were recorded from admission and throughout labour (table 3). Similar to the spontaneous labour group, the percentage of examinations where a purple line was present increased with an increase in cervical dilatation although this number reduced from 80% with a cervical dilatation of 7-8 cms to 52.2% when cervical dilatation was 9-10 cm.

**Table 2:** VEs and examination of line for women in spontaneous labour (n = 112) categorised by cervical dilatation

Cervical Dilatation (cm)	Number (%) of VEs at specific cervical dilatation where line was present	Mean length of line (cm), (SD)	Number of VEs and examination of line
<1	0	-	7

1-2	6 (27.3)	5.3, (3.1)	22

3-4	43 (55.9)	7.8, (3.4)	77*

5-6	54 (71.1)	7.8, (2.5)	76

7-8	32 (70.0)	8.7, (2.2)	46*

9-10	54 (72.0)	9.6, (2.1)	75*

*1 line measurement not recorded			Total 303

**Table 3:** VEs and examination of line for women admitted for induction of labour (n = 32) categorised by cervical dilatation

Cervical Dilatation (cm)	Number (%) of VEs at specific cervical dilatation where line was present	Mean length of line (cm), (SD)	Number of VEs and examination of line
<1	1 (8.3)	12	12

1-2	10 (26.3)	8.3 (1.9)	38

3-4	9 (47.4)	7.8 (2.1)	19*

5-6	6 (75.0)	8.2 (2.9)	8

7-9	4 (80.0)	8.9 (0.3)	5

9-10	12 (52.2)	9.9 (2.8)	23*

*1 line measurement not recorded			Total 105

## Discussion

This study confirms the findings of Bryne and Edmonds [32] that the purple line is present in the majority of women in active labour. In our sample, 76% of women had a measurable purple line at some point in their labour. There was a medium positive correlation between length of the purple line and cervical dilatation and station of the fetal head indicating that as labour progressed, the purple line increased in length. Our data suggests that for over a quarter of the VEs examinations performed where the women's cervical dilatation was between 1-2 cms, a purple line was present. This figure rises to approximately 50% of the VEs at 3-4 cms dilated.

This is the first study to rigorously examine when the line begins to appear and also to observe whether the line is present before labour commences. All examinations of the line were checked by two midwives increasing the confidence in findings. A within subjects correlation analysis was undertaken and weighted according to the number of measurements recorded from each woman. The importance of this type of analysis where several measurements are taken from the same person has been highlighted [37] as the variability of measurements made on different people are usually much greater than the variability between measurements on the same person. It is important to note that as we were interested in whether a change in length of the purple line was paralleled by changes in either cervical dilatation or station of the fetal head, those women with only one measurement of cervical dilatation or length of line were not included in this analysis.

Although our study did not recruit all eligible women, the planned sample size was achieved. The number of women who were approached by the midwife to participate in this study and refused is high. The reasons these women did not wish to participate was not recorded.

Recruiting women to studies of intrapartum care is challenging. Problems such as a reliance on midwives to identify and consent appropriate women, the potential for selection bias as those women in early labour are more likely to be approached, and the problem of gaining full informed consent from women who are often very distressed have been described [38].

In this study, we asked midwives to only recruit those women who were able to give full and informed consent and this therefore led to some women too advanced in labour not being approached. Also, we relied on the midwives to recruit women and collect all data and therefore when the labour ward was busy, midwives reported to either forgetting about the study or that it was not a priority for them.

Bryne and Edmonds [32] postulated that the purple line appeared due to increasing intrapelvic pressure as the fetal head descends causing vasocongestion in the basivertebral and intervertebral veins at the sacrum which along with a lack of subcutaneous tissue in this area resulted in the line of red or purple colouration; however the aetiology of the purple line is unknown. This possible explanation may account for the correlation seen in the present study between station of the fetal head and length of the purple line and its absence before labour starts. However, it is interesting to note that there was no relationship between baby's birth weight and the presence of the line but this may be due to the sample size in the present study.

Where present, the purple line may provide a useful adjunct for clinicians assessing progress in labour, alongside other measures, and may avoid the need for some VEs especially when considering women who decline or find the examination intrusive. It will however, not replace every VE in labour which not only assesses cervical dilatation but also assesses, presentation and position of the fetus, position, effacement and consistency of the cervix and also confirms whether or not the membranes are intact [34].

The data in this study raise some questions which warrant further research. Why did the line not appear in 24% of women and why was the line more likely to appear in women who had a spontaneous labour? It is also interesting to note that in our sample of women admitted for induction of labour, the presence of the line appeared to decrease in those at 9-10 cm dilated. Future research should look closely at the potential confounding variables that may have impacted on the line presence such as use of epidurals, or position in labour. Also, 97% of our sample were Caucasian women and therefore it would be interesting to explore whether the line is also present in women across other ethnic and racial groups. The authors are aware that no measure of inter-rater reliability was conducted in this study and any future research should attempt to measure this. We are also aware that methodologically, it would have been preferred if the measurements of labour progress (cervical dilatation and station of fetal head) and length of the purple line be blinded. This however would have resulted in three different midwives recording this data which would have been impractical and intrusive in terms of the woman's care. Further research may consider examining the width and colour of the line. It is also important to review the acceptability of this examination to both women and midwives.

## Conclusion

This study has shown that the purple line does exist, and there is a medium positive correlation between its length and both cervical dilatation and station of the fetal head. What needs to be considered now is whether this line is clinically useful in the management of labour and whether the assessment and measurement of the line is itself acceptable to not only women in labour but also to clinicians as any potentially reliable measurement of labour progress has to be acceptable to both.

## Competing interests

The authors declare that they have no competing interests.

## Authors' contributions

AS, HC, CM, MS and CN designed the study. AS and HC co-ordinated the study and analysed some of the data. SK co-ordinated enrolment of women to the study and AS, HC, CM, MS and CN contributed to the writing of this paper. All authors read and approved the final draft.

## Pre-publication history

The pre-publication history for this paper can be accessed here:

http://www.biomedcentral.com/1471-2393/10/54/prepub
